# Effects of Photoperiod Change on Melatonin Secretion, Immune Function and Antioxidant Status of Cashmere Goats

**DOI:** 10.3390/ani9100766

**Published:** 2019-10-06

**Authors:** Chenyu Mao, Yuanqing Xu, Lulu Shi, Shiwei Guo, Xiao Jin, Sumei Yan, Binlin Shi

**Affiliations:** College of Animal Science, Inner Mongolia Agricultural University, Hohhot 010018, China; mcyyy1992@163.com (C.M.); happyxyq@yeah.net (Y.X.); 15024913949@163.com (L.S.); 2017202010028@emails.imau.edu.cn (S.G.); yaojinxiao@aliyun.com (X.J.); yansmimau@163.com (S.Y.)

**Keywords:** photoperiod change, melatonin secretion, immune function, antioxidant status, gene expression, goat

## Abstract

**Simple Summary:**

Considering that the photoperiod can affect melatonin (MLT) secretion and MLT can be used as reactive oxygen species scavenger and immunomodulator in animals, the present experiment was designed and conducted to study the effects of photoperiod change on MLT secretion, immune function and antioxidant status of cashmere goats. The results showed that the photoperiod of 8 h light and 16 h dark per day resulted in goats having a higher concentration of MLT and could effectively enhance the immune function and antioxidant enzyme activity of goats.

**Abstract:**

The photoperiod affects animals’ secretion of hormones, especially melatonin (MLT), which is involved in the regulation of the immune function and antioxidant status. The present experiment was conducted to study the effects of the photoperiod on MLT secretion, immune function, antioxidant status and related gene expression in goats. Eighteen adult female cashmere goats were randomly divided into three photoperiod groups: the control group (CG: natural photoperiod); the short-day photoperiod group (SDPP group: 8 h light; 16 h dark) and the shortening-day photoperiod group (SIPP group: lighting time shortened gradually from 16 h/d to 8 h/d). The experiment lasted for 60 days. The results showed that SDPP increased MLT concentration in serum at day 30 of the experiment (*p* < 0.05), but SIPP increased it at day 60 (*p* < 0.05). The activity of total superoxide dismutase (T-SOD), glutathione peroxidase (GPx) and catalase (CAT) increased (*p* < 0.05), and malondialdehyde (MDA) concentration decreased (*p* < 0.05) at day 30 in SDPP; no significant effects of SIPP were observed at day 30. Both SDPP and SIPP goats had higher activities of T-SOD, GPx and CAT (*p* < 0.05) at day 60. The concentration of immunoglobulin G (IgG), interleukin 1β (IL-1β) and interleukin 2 (IL-2) increased in SDPP (*p* < 0.05) at day 30. Both SDPP and SIPP raised the concentration of IgG, IL-1β and IL-2 at day 60 (*p* < 0.05). For the relative gene expression, the SDPP improved the gene expression of *SOD1*, *CAT*, *GPx4*, *nuclear factor erythroid-2-related factor 2(Nrf2)*, *IL-1β*, *IL-2* and *tumor necrosis factor-α (TNF-α)* (*p* < 0.05) in blood leukocytes at day 30. In addition, at day 60, goats in the SDPP group had a higher gene expression of *CAT*, *GPx4*, *IL-1β* and *IL-2* (*p* < 0.05). Goats in SIPP had significantly higher gene expression of *SOD1, CAT, GPx4, Nrf2, TNFα, IL-1β* and *IL-2* (*p* < 0.05) than those in CG. These results indicated that SDPP and SIPP could secrete more MLT and then improve the immune function and antioxidant status of the goats.

## 1. Introduction

Environmental conditions influence livestock activities; for example, temperature can directly stimulate immune response, and the length of illumination can change hormone secretion and alter immune function and antioxidant status. The immune response caused by cold or heat stress has been studied, but there are few reports on immune regulation and antioxidant status variation caused by the photoperiod. Regarding lighting, the light signal conduction comes first. The signal of light is transmitted to the superior chiasmal nucleus of the hypothalamic through the retina, then goes to the pineal gland (PG) through the paraventricular nucleus [1], which is followed by melatonin (MLT) secretion [2]. Then, the PG sends its output signal. MLT regulates the breeding activity via retrograde and anterograde pathways [3,4,5], altering immune function and antioxidant status [6,7].

MLT is a multifunctional molecule whose duration and concentration are mainly affected by the photoperiod, and its main function is alleviating oxidative stress and regulating immune function. It had been reported that daily variation in circulatory MLT level during different seasons could influence the immune system and free-radical scavenging capacity in mammals, including human beings [8]. The effects of MLT are likely mediated via receptor signal transduction as well as anti-oxidative [9,10] and anti-apoptotic mechanisms [11]. MLT is effective in reducing oxidative stress, and exogenous MLT injection enhances the level of glutathione peroxidase (GPx) and total superoxide dismutase (T-SOD) in tissues of mice and chickens [12,13,14], and reduces the level of malondialdehyde (MDA) and increases the activity of catalase (CAT) in the serum of the Chhotanagpuri ewe [15]. On the contrary, pinealectomized rodents reduce the level of glutathione peroxidase (GPx) in several tissues because of a lack of MLT [16]. Also, MLT may directly act on immune cells to release cytokines to produce immunomodulatory effects. It has been shown that MLT increases the production of interleukin 1β (IL-1β), interleukin 2 (IL-2), interleukin 6 (IL-6) and γ-interferon (γ-IFN) in human peripheral blood mononuclear cells (PBMCs) cultured in vitro [17].

However, until now, no information has been available about the effect of MLT change mediated by different photoperiods on the regulation of immune functions and antioxidant status in goats. Thus, our objective was to determine the effect of MLT secretion induced by photoperiod variation on immune function, antioxidant status and related gene expression in cashmere goats.

## 2. Materials and Methods

### 2.1. Ethics

Goats were obtained from the Experimental Farm of Inner Mongolia Agricultural University. All animal experiments were performed in accordance with the national standard Guideline for Ethical Review of Animal Welfare (GB/T 35892-2018).

### 2.2. Animal and Experiment Design

The experiment was conducted in the experimental farm of the Inner Mongolia Agricultural University, Hohhot, Inner Mongolia, China, from 18 April to 16 June, 2018, and lasted for 60 days, during which the natural photoperiod increased. A total of 18 adult female cashmere goats with similar body weight (13.57 ± 0.66 kg) were randomly divided into 3 treatment groups with 6 head per group and housed in 3 environmentally controlled rooms, respectively. Goats in the control group (CG) received the natural photoperiod; those in the short-day photoperiod group (SDPP) received 8 h light and 16h dark per day, with light provided by natural light from 10:00 to 18:00; and those in the shortening-day photoperiod group (SIPP) received 16 h light and 8 h dark initially, then the lighting time shortened gradually by 1 h per week to 8 h light and 16 h dark per day, with light provided by fluorescent lamps when natural light vanished. All 3 groups were fed the same diets at 10:00 and 17:00 which were formulated to meet or exceed the nutrient requirements recommended by the National Research Council (NRC, 2007). Water was provided ad libitum.

### 2.3. Preparation of Blood Sample and Analysis

Blood samples were collected at 5:00 weekly during the first 30 days, and once every 2 days during the last 30 days for the analysis of MLT, whereas for the analysis of immune indexes and antioxidant indexes, the blood samples were collected on day 30 and day 60. The serums were harvested after centrifugation for 20 min at 3000× *g* and then frozen at −20 °C. Leukocytes were harvested and stored in liquid nitrogen for mRNA extraction. MLT concentration and immune indexes, including IgG, IgA, IgM, IL-1β, IL-2 and *tumor necrosis factor-α* (TNF-α) concentration, were determined with commercial ELISA kits (Ruixin Biological Technology Co., Ltd. Quanzhou, China) according to the manufacturer’s instructions. Antioxidant indexes, including total antioxidant capacity (T-AOC), T-SOD, CAT, GPx and MDA, were determined with commercial kits (Nanjing Jiancheng Institute of Bioengineering, Nanjing, China).

### 2.4. Total mRNA Extraction and Quality Determination

Total RNA was obtained using Trizol Reagent according to the manufacturer’s protocol. The extracted mRNA was quantified spectrophotometrically and the OD_260_/OD_280_ was used for evaluation of quality. Subsequently, the total mRNA was treated with DNase I (TaKaRa Biotechnology Co. Ltd., Dalian, China) to remove gDNA, and then reverse-transcribed into cDNA on LifeECO (Bori Technology Co., Ltd. Hangzhou, China) using a Prime Script RT™ Master Mix kit (TaKaRa Biotechnology Co. Ltd., Dalian, China). The reactions were performed with incubation for 15 min at 37 °C, followed by 5 s at 85 °C.

### 2.5. Quantitative RT-PCR Analysis

The resulting cDNAs were used in quantitative RT-PCR (qRT-PCR) reactions. The qRT-PCR for target genes and housekeeping genes (*β-actin*, *B2M* and *YWHAZ*) was performed in triplicate using the LightCycler^®^ 96 Real-Time PCR Design & Analysis System (ROCHE Ltd., Basel, Switzerland) with a SYBR^®^ Premix Ex Taq™ Kit (TaKaRa Biotechnology Co. Ltd., Dalian, China). The qRT-PCR was performed using 20 μL reactions that contained 10 μL of 2 × TB Green Premix Taq II, 0.8 μL of each primer (10 μM), 6.4 μL of water and a 2 μL cDNA template. This was performed with the following cycling conditions: 95 °C for 30 s (hold stage), followed by 40 cycles of 95 °C for 15 s and 60 °C for 30 s (PCR stage), then 95 °C for 10 s, 60 °C for 1 min, and 95 °C for 15 s.

Goat *SOD1*, *SOD2*, *GPx1*, *GPx4*, *nuclear factor erythroid-2-related factor 2 (Nrf2)*, *CAT*, *IL-1β*, *IL-2*, *IL-6*, *TNF-α* primers for quantitative RT-PCR were designed as previously reported (Table 1). The goat *SOD1*, *SOD2*, *Nrf2* primer sequences and information are described by Ma et al. [18], whereas the goat *GPx1*, *CAT* primer sequences are described by Yao et al. [19], and the goat *GPx4* primer sequences are described by Lowe et al. [20]. The goat *IL2*, *IL-6*, *TNF-α* primer sequences and information are described by Zhang [21] and *IL-1β* primer sequences and information are described by Liu et al. [22]. *β-2-microglobulin (B2M)*, *tyrosine 3-monooxygenase (YWHAZ)* and *beta-actin (β-actin)* were treated as housekeeping genes, and are described by Wang [23] (Table 1). The relative quantity of target gene mRNA was expressed as 2^−∆∆ct^ using the relative comparative threshold cycle method as described previously [24], and for the normalization of the RT-qPCR data, the geometric mean Ct of 3 reference genes was used [25].

### 2.6. Statistical Analysis

Data were analyzed by one-way ANOVA using the generalize linear model (GLM) procedure of SAS for Windows (Version 9.4, SAS Institute Inc., North Carolina, NC, USA). Differences among the treatment means were detected using Duncan’s test, and considered significant at *p* < 0.05. Data are presented as mean ± SD.

## 3. Results

### 3.1. MLT Secretion

**Figure 1:**
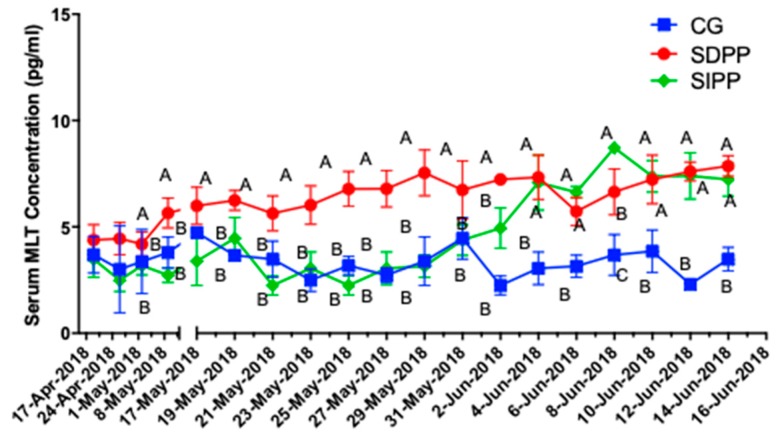
Effect of photoperiod change on MLT secretion. Different letters indicate significant difference at *p* < 0.05 among the groups. CG: control group; SDPP: short-day photoperiod; SIPP: shortening-day photoperiod.

As shown in Figure 1, goats in SDPP group increased MLT concentration at day 28, compared with those in CG and SIPP groups (*p* < 0.05). The MLT concentration of SIPP was higher than that of CG on day 45 (*p* < 0.05). From day 47, the goats in both SDPP and SIPP had a higher MLT concentration (*p* < 0.05) than those in CG.

### 3.2. Immune Function

The effects of photoperiod change on immune system parameters are presented in Table 2. Compared with CG, goats in SDPP increased the concentration of IgG, IL-1β and IL-2 in serum (*p* < 0.05) on day 30, but no significant differences (*p* > 0.05) were observed between CG and SIPP. At day 60, the concentration of IgG and IL-1β in serum increased in both SDPP and SIPP (*p* < 0.05) (Table 2). An increased content of IL-2 was observed on day 60 in SDPP, compared to CG goats (*p* < 0.05).

### 3.3. Antioxidant Status Indicators

The effects of photoperiod change on antioxidant status indicators are presented in Table 3. At day 30, compared with CG goats, SDPP goats had higher activity of T-SOD (*p* < 0.05), CAT, T-AOC and GPx (*p* < 0.01) and lower MDA concentration (*p* < 0.05) in the serum. At day 60, SDPP enhanced the activity of T-SOD, CAT (*p* < 0.01) and GPx (*p* < 0.05) in serum, and lowered the concentration of MDA (*p* < 0.05) compared with those in CG (Table 3).

There was no difference in antioxidant status between SIPP and CG at day 30. However, SIPP goats had higher activity of T-SOD, CAT (*p* < 0.01), and GPx (*p* < 0.05) and lower MDA concentration (*p* < 0.05) in serum on day 60 than goats in CG.

### 3.4. The Relative Expression of mRNA

**Figure 2:**
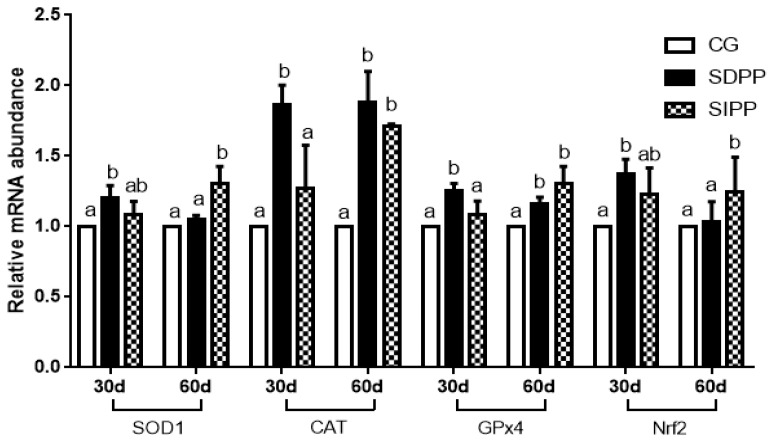
Effect of photoperiod change on relative gene expression of *SOD1*, *CAT*, *GPx4* and *Nrf2*. Bars carrying different letters (a, b) were significantly different (*p* < 0.05) (mean ± standard error, n = 6); CG: control group; SDPP: short-day photoperiod; SIPP: shortening-day photoperiod.

**Figure 3:**
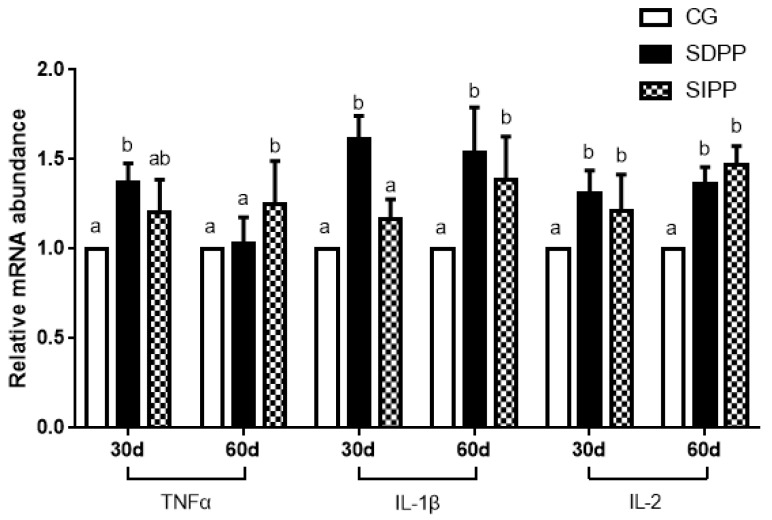
Effect of photoperiod change on relative gene expression of *TNF2*, *IL-1β* and *IL-2*. Bars carrying different letters (a, b) were significantly different (*p* < 0.05) (mean ± standard error, n = 6); CG: control group; SDPP: short-day photoperiod; SIPP: shortening-day photoperiod.

**Figure 4:**
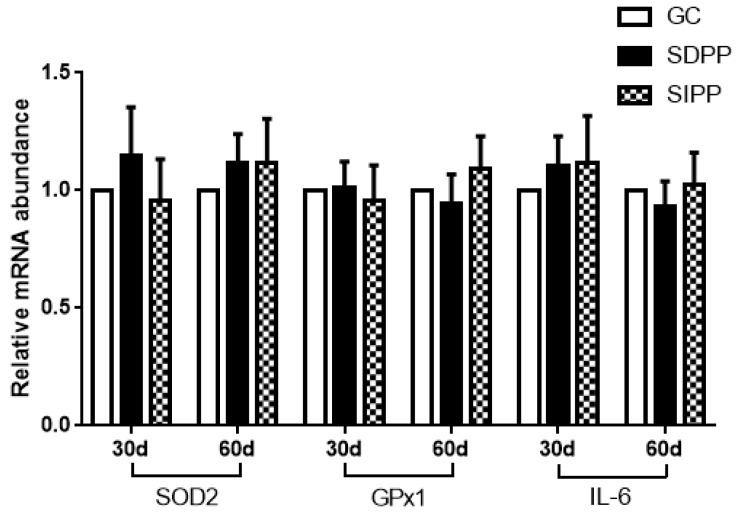
Effect of photoperiod change on relative gene expression of *SOD2*, *GPx1* and *IL-6*; CG: control group; SDPP: short-day photoperiod; SIPP: shortening-day photoperiod.

On day 30, the SDPP improved the gene expression of *SOD1*, *CAT*, *GPx4*, and *Nrf2* (Figure 2), and up-regulated the gene expression of *TNFα*, *IL-1β* and *IL-2* (Figure 3). There was no change in gene expression in SIPP compared with CG. At day 60, the SDPP goats had higher gene expression of *CAT*, *GPx4* (Figure 2), *IL-1β*, *IL-2* (Figure 3) (*p* < 0.05) than CG. Meanwhile, the SIPP increased the gene expression of *SOD1*, *CAT*, *GPx4* and *Nrf2* (Figure 2), *TNF-α*, *IL-1* and *IL-2* (Figure 3) (*p* < 0.05) on day 60 compared with CG. The photoperiod change had no effect on gene expression of *SOD2*, *GPx1* and *IL-6* (Figure 4).

## 4. Discussion

In the present study, we determined the effect of the photoperiod on MLT secretion, immune function and antioxidant status of goats. For this, we used three models of photoperiod, which were nature photoperiod, short-day photoperiod, and shortening-day photoperiod, respectively. Our results confirmed that when the photoperiod was 8 h light and 16 h dark, the goats had a higher MLT secretion, immune function and antioxidant status. Moreover, our results showed, as the illumination duration decreased, the MLT secretion, immune function and antioxidant status of the goats gradually increased, which might explain why the temperature in winter was low, but the immune function did not decrease.

The light signal is converted into a neural message on the retina, and then the signal is transmitted to the optic nerve of the hypothalamus. Except for the optic nerve, the multiple central and peripheral sympathetic neurons also transmit information to the pineal gland [26]. At night, an electrical signal is transmitted to the pineal gland, which causes norepinephrine to be released from the post-ganglionic sympathetic nerve terminals into the pineal gland, maintaining MLT mainly through the β1 adrenergic receptor [27]. Many studies have shown that photoperiod change directly affects the secretion of MLT, and induces significant circadian rhythm change [28]. The duration of MLT at a high level at nighttime represents the length of the night, which can signal seasonal changes and regulate seasonal activities [29].

The concentration of MLT was affected by photoperiod significantly, and then altered immune function and antioxidant status. Molecular oxygen is important for the survival of all aerobic organisms. O_2_ acts as the final electron acceptor in the process of aerobic energy metabolism and is reduced to H_2_O by mitochondrial enzymes in the cell. However, when the organism is subjected to endogenous and exogenous stimuli, the partially reduced and highly active metabolites of O_2_ may form superoxide anion (O_2_-) and hydrogen peroxide (H_2_O_2_). These reactive oxygen species (ROS) are highly reactive and toxic, and can cause an increase in oxidative stress in various tissues [30]. Oxidative stress can damage biomolecules, including cellular lipids, proteins, amino acids, and deoxyribonucleic acid, thereby inhibiting their normal function [31,32]. The main defense against superoxide anion radical damage and its active product damage is the superoxide dismutase enzyme (SOD) series [33]. Studies showed that the daily variation of antioxidant enzyme activity in serum of Indian goats was related to MLT concentration both in winter and summer [8]. Similarly, Albarran et al. [34] found SOD activity had a parallel reaction with the MLT level in winter. This direct correlation of MLT and SOD enzyme activity in different seasons has also been reported in different animal tissues and at the circulating level. In addition to seasonal differences, antioxidant activity also has a circadian rhythm, which is closely related to the circadian rhythm of MLT [35,36]. This illustrated that changes in physiological levels of melatonin are adequate to alter the antioxidative defense system as reflected in the activities of antioxidative enzymes.

In addition, the other two antioxidant enzymes, catalase (CAT) and glutathione peroxidase (GPx) participate in the removal of H_2_O_2_ from the cellular environment. CAT, present mainly in the peroxisomes, presents a molecule of ferric ion at its active site and converts two molecules of H_2_O_2_ into one molecule of water and diatomic oxygen [37]. Interestingly, CAT activity had a parallel relationship with the pattern of MLT levels. Previous experiments showed that exogenous MLT could increase the activity of CAT in red blood cells of Wistar Albinos rats exposed to aluminum sulfate and the activity increased with increased MLT concentration [38]. Additionally, other experiments showed that MLT could increase the concentration of CAT in the blood of rats, which was consistent with the results of the present study [39]. GPx is an important peroxide-degrading enzyme widely present in the body. GPx can catalyze the conversion of GSH to oxidized glutathione (GSSG) and reduce toxic peroxides to non-toxic hydroxy compounds, thereby protecting the structure and function of cell membranes from peroxide interference and damage. In the present study, GPx concentration increased at day 30 in SDPP and was maintained at a high level until the later stage of the experiment, which indicated dose dependence on the concentration of MLT, and the phenomenon was confirmed in SIPP. It has been proved that exogenous MLT injection increases GPx concentration in the plasma of hamsters [40] and relieves oxidative stress induced by doxorubicin and increases the concentration of GPx in the heart of rats [41]. However, pinealectomized rats without MLT injection reduced GPx activity levels in several tissues [16]. Furthermore, continuous exposure to light is known to reduce night-time GPx activities in chicks, which is associated with nocturnal MLT decline [42].

MDA is the most familiar breakdown product of lipid peroxides, and is considered to be one of the reliable markers of cellular peroxidative damage [43]. It had been demonstrated that MLT could curb the cytotoxic effects induced by MDA [44] and reduce lipid peroxidation in mammalian cells [45]. In our study, when the concentration of MLT in serum was high, the content of MDA was lowered, suggesting that MLT could depress lipid peroxidation. Previous in vitro study on human PBMCs incubated with different doses (10^−5^ M–10^−9^ M final concentration) of MLT showed that the MDA content had a dose-dependent relationship with MLT, i.e., a higher concentration of MLT caused a lower content of MDA [46]. Moreover, MLT reduced the content of MDA in red blood cells and the kidney of acute organophosphorus poisoned rabbits [47]. The protection of lipids by MLT can be explained as a preventive antioxidant in our system. On one hand, MLT has the ability to extinguish free radicals initiating lipid peroxidation [48]. On the other hand, its secondary and tertiary metabolites are able to neutralize numerous toxic oxygen derivatives, which might cause lipid peroxidation [49]. In addition, the block of lipid peroxidation mediated by MLT could be ascribed to a concomitant increase of antioxidant enzyme activity.

T-AOC, which includes a number of antioxidant enzymes and related biomolecules that can remove free radicals from a specific organ or living organism, reflects the total antioxidant ability [43]. In the present study, goats in SDPP had a higher T-AOC at the middle of the trial, but in other stages of the trial, T-AOC did not differ among the three treatment groups. However, Subramanian et al. [44] explored the antioxidant role of MLT in rats and observed that the peak of T-AOC was closely synchronized with the nocturnal peak of MLT. Moreover, in a study of chicks, Albarran et al. [34] found that, with an increased MLT level in serum, the T-AOC level in serum, lung and kidney also increased. The reason in present study could be ascribed to the homeostatic mechanism or biological oxidative balance [46], and requires further experiments for explanation.

MLT secreted by the pineal gland plays an important role in many physiological functions and is an effective free radical scavenger [50,51]. Previous studies showed that MLT could activate the Nrf-2/ARE antioxidant pathway against the external environment or UV-induced damage, which suggests this pathway is one of the protective mechanisms [52,53]. Nrf2 is a vital transcription factor that modulates redox responses to manage oxidative stress [54]. The expression levels of antioxidant enzymes including *SOD*, *CAT*, and *GPx*, as well as GSH synthesis, were induced by the binding of *Nrf2* to antioxidant response element [55]. According to previous studies, MLT was found to attenuate brain injury and regulate the expression of antioxidant enzyme (AOE) genes in an experimental model of traumatic brain injury by activating the *Nrf2* pathway [56]. Also, in the same pattern, MLT was found to regulate the expression of AOE genes in the cerebral cortex and lacrimal gland under porphyrin-induced damage. In the present study, the results showed MLT could induce the expression of *Nrf2*, *SOD*, *CAT* and *GPx* genes under non-oxidative stress, and increase the concentration of SOD, CAT and GPx. This may support the hypothesis that MLT acts as an antioxidant in a variety of ways, such as concentration or duration, which can induce related gene expression, enhance related enzyme activity, or act directly as a ROS scavenger.

Serum immunoglobulin is the most persistent and important antibody in the primary immune response, and is used to promote phagocytosis of monocytes and macrophages, neutralize the toxicity of bacterial toxins, and combine with viral antigens to prevent the virus infecting host cells [43]. Negrette et al. [57] found exogenous MLT could enhance the body’s humoral immunity by enhancing IgM concentration. In addition, Akbulu et al. [58] found that exogenous MLT injection could promote the increase of serum IgG and IgM levels in 28-month-old Wistar rats. Similarly to the current findings, Demas et al. [59] found that the serum IgG level in mice in a short-light low-temperature group was higher than in mice in a long-light high-temperature group.

ILs are a group of cytokines that play an important role in the immune system. Among them, IL-1β mediates the immune response by (1) promoting the proliferation and differentiation of thymocytes and mature T-cells; (2) enhancing B-cell differentiation; (3) inhibiting the growth of tumor cells and killing them; and (4) inducing T-cells to generate IL-2 [60]. IL-2 is a key cytokine with broad immunomodulatory activity [60]. In the present study, results suggested that MLT might directly act on immune cells to release cytokines to produce immunomodulatory effects. According to Pioli, MLT could promote the production of IL-1 and TNF-α in mouse spleen macrophages, and their contents in a test group were 2.2 times and 1.2–4.8 times those of the control group, respectively [61]. Additionally, the level of plasma IL-2 in pinealectomy mice was significantly decreased, but after treatment of exogenous MLT injection, the content of IL-2 increased [62]. TNF-α, another cytokine, usually shows a circadian rhythm and has a high level at night, possibly affected by MLT [63]. Administration of exogenous MLT in the morning showed TNF-α level in a general upregulation [64].

In recent years, many studies reported the existence of a relationship between MLT and the immune system in animals and humans. In 1973, Vaughan et al. [65] found that short light could increase the thymus weight of voles. Since then, many reports confirmed that MLT peaks were associated with the number of lymphocytes in the blood, and these studies have been carried out in tropical palm squirrels and humans [66,67]. Moreover, pinealectomized animals will reduce their immune function, and this theory has been confirmed in mice [68,69]. An additional function of MLT in the immune system is to regulate gene expression of cytokines in the spleen, thymus, and lymph nodes. In C57 mice, MLT up-regulated the level of gene expression of *TNF-α*, transforming growth factor-beta, and stem cell factor *(SCF)* in peritoneal macrophages, and the level of *IL-1β*, *TNF-α*, and *SCF* in splenocytes [70]. Similarly, in the present study, MLT up-regulated the level of gene expression of *IL-1β, IL-2*, and *TNF-α*.

## 5. Conclusions

In conclusion, the photoperiod variation had effects on MLT secretion, immune function and antioxidant status in cashmere goats. The short-day condition triggered the high level of MLT that was responsible for a high level of immune function and antioxidant status. In addition, the related gene expression was up-regulated by the high level of MLT caused by the change of photic signal, which emphasized the role of MLT as an antioxidant. Therefore, it can be suggested that improvement in the level of MLT by exposing animals to short days may improve the immunity of livestock. Further experiments will be needed to identify the underlying molecular mechanism, which can help determine how MLT influences the immune system and antioxidant status.

## Figures and Tables

**Table 1:** Primers for quantitative real-time PCR.

Target		Sequence of Nucleotide (5′-3′)	Genebank No.	Size (bp)
*SOD1*	F	ATCCACTTCGAGGCAAAGGG	NM_001285550.1	104
	R	GCACTGGTACAGCCTTGTGTA		
*SOD2*	F	TCAATAAGGAGCAGGGACGC	XM_005684984.1	85
	R	AGCAGGGGGATAAGACCTGT		
*GPx1*	F	ACATTGAAACCCTGCTGTCC	XM_005695962.2	216
	R	TCATGAGGAGCTGTGGTCTG		
*GPx4*	F	TTCCCTTGCAACCAGTTTGG	NC_030814.1	105
	R	TCATCCATTTCCACAGAGGGT		
*Nrf2*	F	AGCCAGGTGAGATGGAACTG	XM_005679848.2	120
	R	CCAGACTCCCTGTTTCGCTG		
*CAT*	F	CACTCAGGTGCGGGATTTCT	GQ_204786.1	159
	R	ATGCGGGAGCCATATTCAGG		
*IL-1β*	F	CATGTGTGCTGAAGGCTCTC	D63351.1	173
	R	AGTGTCGGCGTATCACCTTT		
*IL-2*	F	TGTCTTGCATTGCACTAACTCTTGC	AF_307018.1	116
	R	CCCAAAAGCAACTGTAAATCCAGC		
*IL-6*	F	GGGCTGCTCCTGGTGATGACTT	HM_565937.1	133
	R	CGATGTGCTTAATGAGAGCTTCGG		
*TNF-α*	F	CAACAGGCCTCTGGTTCAGAC	NC_030830.1	209
	R	GGACCTGCGAGTAGATGAGG		
*β-actin*	F	ACTGGGACGACATGGAGAAGA	U39357	199
	R	GCGTACAGGGACAGCACAG		
*B2M*	F	GGTGCTGCTTAGAGGTCTCG	NM_001009284	109
	R	ACGCTGAGTTCACTCCCAAC		
*YWHAZ*	F	TGTAGGAGCCCGTAGGTCATCT	AY970970	102
	R	TTCTCTCTGTATTCTCGAGCCATCT		

*B2M* = β-2-microglobulin; *YWHAZ* = tyrosine 3-monooxygenase; *β-actin* = beta-actin; F: Forward primer; R: Reverse primer.

**Table 2:** Effects of photoperiod change on immune system parameters.

Day	Item	Groups			*p*-Value
		CG	SDPP	SIPP	
30 d	IgG (mg/mL)	211.8 ± 18.4 ^a^	264.9 ± 10.8 ^b^	220.7 ± 20.8 ^a^	0.043
	IgM (μg/mL)	160.8 ± 19.6	158.8 ± 22.9	165.8 ± 28.7	0.631
	IgA (μg/mL)	86.97 ± 12.84	94.83 ± 15.86	88.57 ± 19.21	0.427
	IL-1β (ng/mL)	148.3 ± 10.9 ^a^	190.5 ± 22.6 ^b^	160.3 ± 16.3 ^a^	0.037
	IL-2 (pg/mL)	197.8 ± 15.4 ^a^	247.1 ± 13.1 ^b^	221.4 ± 15.5 ^a^	0.05
	TNF-α (ng/mL)	50.11 ± 8.43	53.85 ± 9.10	52.28 ± 7.36	0.533
60 d	IgG (mg/mL)	202.6 ± 20.9 ^a^	270.8 ± 30.5 ^b^	257.3 ± 15.4 ^b^	0.049
	IgM (μg/mL)	158.9 ± 17.3	163.6 ± 21.8	162.6 ± 19.4	0.738
	IgA (μg/mL)	90.33 ± 5.34	86.31 ± 12.72	83.92 ± 20.95	0.421
	IL-1β (ng/mL)	155.4 ± 12.6 ^a^	186.4 ± 12.8 ^b^	179.3 ± 10.1 ^b^	0.05
	IL-2 (pg/mL)	220.7 ± 19.8 ^a^	269.3 ± 13.9 ^b^	261.3 ± 24.6 ^ab^	0.044
	TNF-α (ng/mL)	44.24 ± 9.21	50.34 ± 7.39	49.34 ± 6.35	0.342

^a,b^ Different superscripts within each row indicate significant differences (*p* < 0.05). CG: control group; SDPP: short-day photoperiod; SIPP: shortening-day photoperiod; IgG: immunoglobulin G; IgM: immunoglobulin M; IgA: immunoglobulin A; IL-1β: interleukin 1β; IL-2: interleukin 2; TNF-α: tumor necrosis factor α.

**Table 3:** Effects of photoperiod change on antioxidant status indicators.

Day	Item	Groups			*p*-Value
		CG	SDPP	SIPP	
30 d	T-SOD (U/mL)	125.0 ± 1.9 ^a^	139.1 ± 8.4 ^b^	126.8 ± 2.3 ^a^	0.05
	GPx (U/mL)	142.7 ± 4.6 ^a^	160.9 ± 5.3 ^b^	137.1 ± 9.4 ^a^	<0.01
	CAT (U/mL)	2.22 ± 0.29 ^a^	3.00 ± 0.23 ^b^	2.37 ± 0.20 ^a^	<0.01
	MDA (nmol/mL)	2.14 ± 0.11 ^a^	1.46 ± 0.21 ^b^	2.38 ± 0.12 ^a^	0.047
	T-AOC (U/mL)	0.243 ± 0.014 ^a^	0.272 ± 0.019 ^b^	0.211 ± 0.023 ^a^	<0.01
60 d	T-SOD (U/mL)	123.7 ± 2. 7 ^a^	143.8 ± 10.4 ^b^	136.3 ± 6.4 ^b^	<0.01
	GPx (U/mL)	146.2 ± 2.0 ^a^	160.1 ± 5.1 ^b^	168.4 ± 5.1 ^b^	0.05
	CAT (U/mL)	2.08 ± 0.16 ^a^	3.68 ± 0.14 ^b^	3.33 ± 0.15 ^b^	<0.01
	MDA (nmol/mL)	2.47 ± 0.33 ^a^	1.45 ± 0.15 ^b^	1.17 ± 0.12 ^b^	0.023
	T-AOC (U/mL)	0.231 ± 0.037	0.259 ± 0.013	0.226 ± 0.011	0.772

^a,b^ Different superscripts within each row indicate significant differences (*p* < 0.05). CG: control group; SDPP: short-day photoperiod; SIPP: shortening-day photoperiod; T-SOD: total superoxide dismutase; GPx: glutathione peroxidase; CAT: catalase; MDA: malondialdehyde; T-AOC: total antioxidant capacity.
